# Tumor-fibroblast interactions stimulate tumor vascularization by enhancing cytokine-driven production of MMP9 by tumor cells

**DOI:** 10.18632/oncotarget.16022

**Published:** 2017-03-08

**Authors:** Michelle Limoge, Alfiya Safina, Amy Beattie, Lauren Kapus, Alexander M. Truskinovsky, Andrei V. Bakin

**Affiliations:** ^1^ Department of Cancer Genetics, Roswell Park Cancer Institute, Buffalo, New York, USA; ^2^ Department of Pathology, Roswell Park Cancer Institute, Buffalo, New York, USA; ^3^ Department of Cell Stress Biology, Roswell Park Cancer Institute, Buffalo, New York, USA; ^4^ University at Buffalo School of Medicine, Buffalo, New York, USA

**Keywords:** fibroblasts, matrix metalloproteinase-9, tumor angiogenesis, TGF-beta, breast cancer

## Abstract

Advance-stage breast carcinomas include significant amounts of fibroblasts and infiltrating immune cells which have been implicated in tumor growth, recurrence, and response to therapy. The present study investigated the contribution of fibroblasts to tumor growth using direct tumor-fibroblast co-cultures and tumor xenograft models. Our findings revealed that fibroblasts enhance breast carcinoma growth by promoting the tumor vasculature *via* the MMP9-dependent mechanism. In tumor-fibroblast co-cultures, fibroblasts increased expression of TGF-β, TNF, and IL-1β cytokines in tumor cells. These cytokines cooperatively induced expression of matrix metalloproteinase MMP9 in tumor cells. Knockdown of MMP9 by shRNA significantly reduced tumor vascularization induced by fibroblasts. Mechanistically, our findings argue that expression of MMP9 in tumor cellsis regulated by crosstalk of TGF-β with TNF and/or IL-1β cytokines. The mechanism of this cooperative response did not involve cross-activation of the canonical signaling pathways as TGF-β did not activate RELA/p65 signaling, while TNF did not affect SMAD signaling. Instead, TGF-β and TNF cytokines co-stimulated MAP kinases and expression of JUN and JUNB, AP1 transcription factor subunits, which together with RELA/p65 were essential for the regulation of MMP9. Depletion of JUN and JUNB or RELA in tumor cells blocked the cooperative induction of MMP9 by the cytokines. Thus, our studies uncovered a previously unappreciated role of tumor-fibroblast interactions in the stimulation of tumor angiogenesis, and an essential role of the MAPK-AP1 axis in the cooperative up-regulation of the angiogenic driver MMP9 by cytokine crosstalk.

## INTRODUCTION

Breast cancer is the second leading cause of cancer-related death among women in North America [1]. In the past decade it has become evident that knowledge of tumor-intrinsic genomic alterations is not sufficient for defining patients with a higher risk of disease recurrence and metastasis. Accumulating data implicates the tumor microenvironment (TME) in breast cancer progression, recurrence, and response to therapy. The TME includes blood vessels, infiltrating stromal (fibroblasts, adipocytes) and immune cells [2]. The interaction between these major cellular components of the breast tumor microenvironment is still poorly understood.

Tumor-associated fibroblasts (TAFs) are the predominant cell type in the TME [3, 4]. Solid evidence supports the hypothesis that TAFs are actively involved in tumor initiation, progression, and metastasis [3]. Experimental evidence indicates that TAFs promote recruitment of pro-tumorigenic immune cells to the tumor by producing pro-inflammatory cytokines [3, 5]. Tumor-infiltrating immune cells can promote tumor growth and metastasis while other immune cells can mount an anti-tumor immune response and eliminate tumor cells [2]. Tumor-associated myeloid cells, such as macrophages and neutrophils (TAMs and TANs), have been linked to the growth of blood vessels, metastasis, and modulation of response to therapy [6]. Breast tumors can also promote expansion and recruitment of immune-suppressive cells such as myeloid-derived suppressor cells (MDSCs) [7]. Recent studies have revealed that myeloid cells contribute to tumor vascularization *via* the angiogenic activity of matrix metalloproteinase MMP9/gelatinase-B and by differentiating into endothelial cells [7–9]. MMP9 stimulates tumor vasculature by releasing and/or activating matrix-deposited pro-angiogenic growth factors, such as VEGF, thereby recruiting vasculature-forming endothelial cells [8] and pericytes [10, 11]. Deletion of MMP9 in myeloid cells abolishes their ability to promote tumor growth [9]. In the TME, MMP9 may also be produced by fibroblasts [10, 12] or breast carcinoma cells [13–15]. In fact, knockdown of MMP9 in metastatic breast carcinoma cells significantly reduces tumor vasculature [13]. Whether tumor and fibroblast cells in the breast TME cooperate in the regulation of MMP9-driven tumor vasculature is currently unknown.

Transforming growth factor-β (TGF-β) and pro-inflammatory cytokines tumor necrosis factor (TNF) and interleukin 1β (IL-1β) have been implicated in the regulation of MMP9 [15–18]. These cytokines are elevated in malignant breast tumors and can be expressed by all cellular components of the TME [19–22]. Ligation of TGF-β cytokines to the serine/threonine kinase type I and type II receptor complex activates canonical signaling by SMAD transcription factors and auxiliary signaling of mitogen-activated protein kinases (MAPKs) and Akt kinase [23–25]. TNF and IL-1β cytokines activate MAPK signaling and canonical NF-κB (nuclear factor kappa-light-chain-enhancer of activated B cells) signaling [26, 27]. NF-κB transcription factor is essential for the pro-inflammatory cytokine-mediated expression of MMP9 [16]. TGF-β-induced expression of MMP9 in breast cancer cells requires TGF-β-activated kinase 1 (TAK1) [17, 18]. TAK1 is also critical for TNF and IL-1β-stimulated activation of the canonical NF-κB transcription factor, consisting of RELA/p65 and NFKB1/p50 subunits [28]. Although TAK1 is involved in the TGF-β and TNF/IL-1β cytokine pathways, the biological functions of these cytokines are largely antagonistic. TGF-β exhibits an anti-inflammatory function [29], in part by antagonizing pro-inflammatory responses to the IL-1β and TNF cytokines [30–33]. On the other hand, pro-inflammatory cytokines suppress TGF-β-mediated responses [34–36]. Thus, the molecular mechanism and the role of TAK1 in the interaction of the TGF-β and TNF/IL-1β cytokine signaling pathways in the breast TME needs further investigation.

The current study explores the contribution of fibroblasts to tumor growth using tumor-fibroblast co-cultures and tumor xenograft models. This study reveals that tumor-associated fibroblasts enhance tumor growth, promoting the formation of tumor blood vessels *via* a mechanism requiring MMP9. In tumor-fibroblast co-cultures, fibroblasts stimulate production of MMP9 by tumor cells. We further provide evidence that MMP9 regulation involves a cooperative interaction of TGF-β and pro-inflammatory cytokines, such as TNF and IL-1β. The mechanism of this cooperative response does not involve cross-activation of the canonical signaling pathways. Instead, TGF-β and TNF cytokines co-stimulate AP1 transcription factor components. Depletion of AP1 components blocks cytokine-mediated induction of MMP9 in tumor cells. Thus, our studies reveal a previously unappreciated role of tumor-fibroblast crosstalk in stimulation of tumor vasculature by the cooperative up-regulation of the angiogenic driver MMP9 *via* a mechanism requiring AP1 transcription factor.

## RESULTS

### Tumor-associated fibroblasts enhance tumor growth

**Figure 1:**
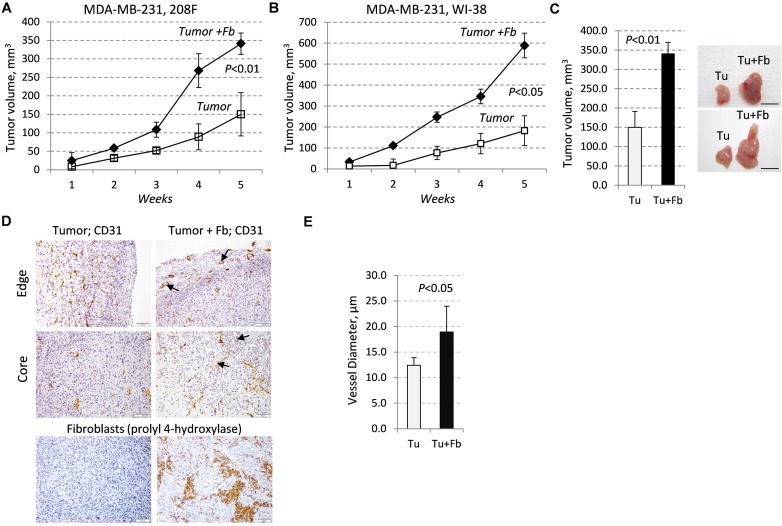
Tumor growth and vasculature are enhanced by fibroblasts **A**.-**B**., Growth of subcutaneous xenografts of MDA-MB-231 cells (empty-vector control, EGFP) ±208F (A) or WI-38 (B) fibroblasts (3:1 ratio) in female SCID mice. **C**. Tumor volume and images at the endpoint of the study with MDA-MB-231-EGFP cells ± 208F fibroblasts. **D**. CD31 (blood vessels) or rPH, prolyl 4-hydroxylase, (rat fibroblasts) staining at the periphery (edge) and core regions of the tumor and tumor-fibroblast mixed xenografts with MDA-MB-231-EGFP cells and 208F fibroblasts; arrows indicate vessels with large lumen. **E**. Quantification of blood-vessel diameters in tumor and tumor-fibroblast xenografts. Vessel diameters were measured at 400× magnification on slides immune-stained for CD31.

In the breast TME, fibroblasts [10, 12] and breast carcinoma cells (13) may produce TGF-β cytokines and MMP9 thereby contributing to tumor vascularization. In this study, we asked whether fibroblasts cooperate with breast cancer cells in tumor growth and vasculature, and whether this effect is linked to MMP9-driven vascularization. Metastatic breast carcinoma MDA-MB-231 cells exhibit autocrine TGF-β signaling [25] that contributes to MMP9-driven tumor angiogenesis [13]. MDA-MB-231 cells were implanted subcutaneously into immune-deficient SCID mice alone or in combination with embryonic female rat 208F fibroblasts. Fibroblasts enhanced the growth of breast carcinoma xenografts by nearly 2.5-fold (Figure 1A), although fibroblasts alone did not form tumors (data not shown). Similar results were obtained with the combination of MDA-MB-231 and human female embryonic WI-38 fibroblasts (Figure 1B). At the endpoint, the mean volume of the tumor-fibroblast xenografts was 2.3 times greater than the tumor xenografts alone (Figure 1C). Immunohistochemical (IHC) analysis with a rat specific marker prolyl 4-hydroxylase β (rPH) confirmed the presence of rat fibroblasts in tumor xenografts at the endpoint of the study (Figure 1D).

Next, we examined whether fibroblasts affect blood vessels. Staining of tumor xenografts with anti-CD31/PECAM1 revealed that fibroblasts markedly enhanced the vessel size at the tumor periphery and in the tumor core (Figure 1D-1E). The presence of blood vessels with a large diameter was found primarily in the tumor-fibroblast xenografts (Figure 1D), while blood vessel densities at the tumor periphery were comparable in both cases (data not shown). Quantification of CD31-stained vessels showed that the mean diameter of the blood vessels was 1.5-fold larger in the tumor-fibroblast xenografts compared to the tumor alone xenografts (Figure 1E). We then examined whether fibroblasts alter tumor cell death. TUNEL (terminal deoxynucleotidyl transferase (TdT) dUTP Nick-End Labeling) staining showed the presence of larger necrotic areas in the core of tumor-alone xenografts whereas no difference between the groups was found at the tumor periphery (Supplementary Figure 1A). We also found that fibroblasts did not increase the proliferative Ki-67 index (data not shown). Together, these results indicate that fibroblasts enhance tumor growth, in part, by promoting tumor vascularization.

### Tumor-fibroblast crosstalk regulates MMP9

**Figure 2:**
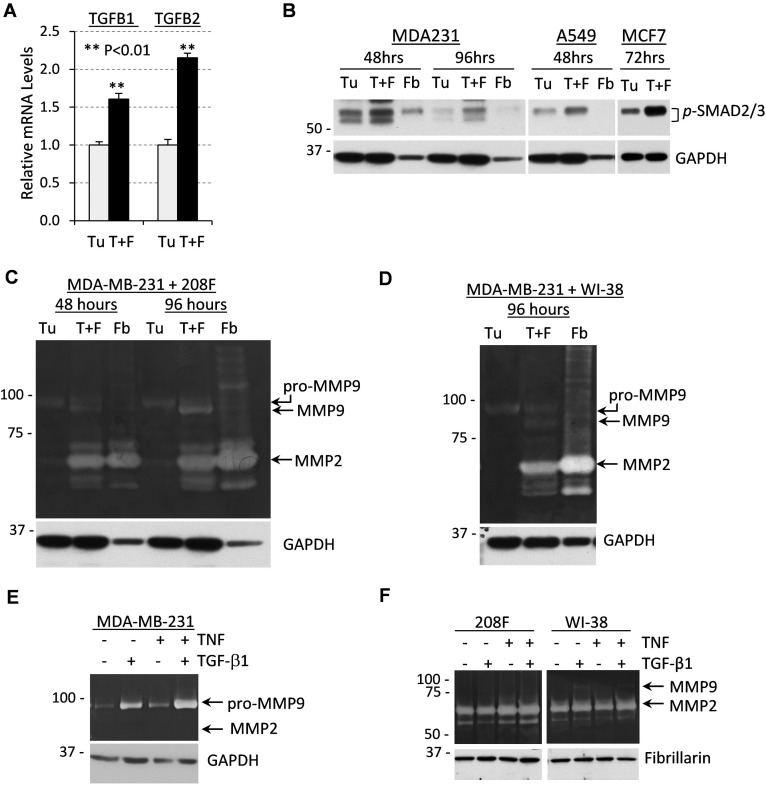
Tumor-fibroblast co-cultures produce high levels of TGF-β cytokines and MMP9 secreted by tumor cells **A**. Relative mRNA levels of TGFB1 and TGFB2 quantified by qRT-PCR using total RNA from MDA-MB-231 cells (Tu) or co-cultures of MDA-MB-231 and 208F rat fibroblast cells in a 3:1 ratio (T+F) for 72 hours. **B**. Whole-cell lysates from co-cultures of MDA-MB-231, A549 or MCF7 tumor cells (Tu) ± 208F or WI-38 fibroblasts (T+F) immunoblotted for TGF-β signaling targets phospho-SMAD2/3. GAPDH was used as a loading control. **C**. Gelatin zymography of 48- or 96-hour conditioned media from MDA-MB-231 cells (Tu), 208F rat fibroblasts (Fb) or their co-culture in a 3:1 ratio (T+F). Lower panel shows immunoblotting for GAPDH. **D**. Gelatin zymography of 96-hour conditioned media from MDA-MB-231 cells (Tu), WI-38 human fibroblasts (Fb) or their co-culture in a 3:1 ratio (T+F). Lower panel shows immunoblotting for GAPDH. **E**. Gelatin zymography with 48-hour conditioned media from MDA-MB-231 cells treated with 2 ng/mL TGF-β1, 10 ng/mL TNF or their combination. GAPDH is a loading control. **F**. Gelatin zymography of 48-hour conditioned media from 208F or WI-38 fibroblasts treated as in (E). Fibrillarin was used as a loading control.

To better understand how fibroblasts promote tumor vascularization, we examined the expression of TGF-β cytokines in direct tumor-fibroblast co-cultures *in vitro*. The microscopic appearance of these co-cultures of tumor and fibroblast cells mimicked the tumor-fibroblast xenografts (Supplementary Figure 1B). Quantitative RT-PCR analysis showed increased mRNA levels of TGF-β cytokines in tumor-fibroblast co-cultures (Figure 2A). To test whether cytokine signaling was also increased, we probed whole-cell extracts with antibodies to phospho-SMAD2/3, a marker of TGF-β signaling. Immunoblotting revealed a significant increase in phospho-SMAD2/3 levels in lysates of 48-hr and 96-hr co-cultures of breast cancer MDA-MB-231 cells with 208F fibroblasts (Figure 2B). Similar results were obtained in co-cultures of lung cancer A549 or breast cancer MCF7 cell lines with fibroblasts (Figure 2B).

Next, we examined the secretion of MMP9 in fibroblast and tumor cells alone, as well as in co-cultures using in-gel gelatin zymography assays. Zymography assays with 48- and 96-hr conditioned media revealed that breast cancer cells alone secrete a 92kDa zymogen form of MMP9 (pro-MMP9), whereas fibroblasts do not produce MMP9 but secrete significant amounts of MMP2/gelatinase-A (Figure 2C). Tumor-fibroblast co-cultures secreted an active 85kDa form of MMP9 that accumulated over the time of incubation (Figure 2C). Similar results were obtained in co-cultures of tumor cells with WI-38 fibroblasts (Figure 2D) as well as in co-cultures of A549 and MCF7 cells with WI-38 fibroblasts (Supplementary Figure 2).

We then asked whether expression of MMPs and cytokines in human breast cancers reflected our findings in cell culture and xenograft studies. Analysis of public data using the *Oncomine* platform (www.oncomine.org) revealed that MMP9 expression is significantly increased in breast carcinomas (Supplementary Figure 4A). Data from a study that isolated RNA from breast tumors using laser capture microdissection (LCM) revealed an 8-fold increase in MMP9 levels (Supplementary Figure 3A, Richardson Breast, [37]). The levels of MMP9 and MMP2 were also increased in the stroma adjacent to invasive breast carcinomas (Supplementary Figure 3B, Finak Breast [38]). Given that MMP9 is regulated by TGF-β and pro-inflammatory TNF/IL-1β cytokines, expression of these cytokines was also examined in human breast carcinomas. Metadata analysis showed a significant increase in expression of these cytokines in both the tumor and stromal compartments of human breast carcinomas (Supplementary Figure 3C). These data are consistent with our experimental findings.

Taking into account these expression data, we examined whether TNF and TGF-β cytokines would cooperate in the regulation of MMP9. Breast cancer cells and fibroblasts were treated with the cytokines alone or in combination, and the conditioned media samples were analyzed for gelatinase activity. In breast cancer cells, expression of MMP9 was stimulated by individual cytokines and this was further enhanced when cells were treated with both cytokines concurrently (Figure 2E). In contrast, MMP9 was not induced by cytokine treatments in two tested fibroblast cell lines (Figure 2F), although both cell lines showed signaling responses to both TNF and TGF-β1 cytokines (Supplementary Figure 4A, shown for 208F). Together these results suggest that tumor-fibroblast co-cultures stimulate expression of cytokines which act upon tumor cells to co-operatively up-regulate MMP9 levels.

### TGF-β but not BMP-type cytokines cooperate with inflammatory cytokines in MMP9 regulation

**Figure 3:**
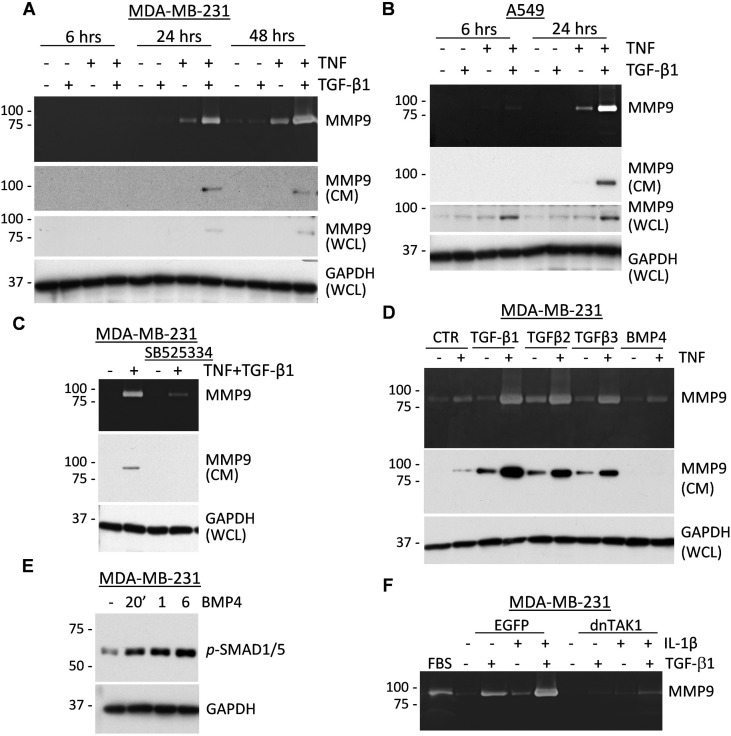
TNF and TGF-β-family cytokines co-regulate secretion of MMP9 **A**.-**B**. Top panels show gelatin zymography of conditioned media from MDA-MB-231 or A549 tumor cells treated with 2 ng/mL TGF-β1 or 10 ng/mL TNF or their combination for the indicated times. Two middle panels show immunoblotting of MMP9 in conditioned media (CM) or whole-cell lysates (WCL). GAPDH is a loading control. **C**. Top panel shows gelatin zymography of 48-hour conditioned media from MDA-MB-231 cells treated with 2 ng/mL TGF-β1 and 10 ng/mL TNF ± 10 μM SB525334, an ALK5/TGFBR1 inhibitor. Bottom panels show immunoblotting of MMP9 in CM and GAPDH in WCL. **D**. Top panel shows gelatin zymography of 48-hour conditioned media from MDA-MB-231 cells treated with 4 ng/mL TGF-β1, 160 ng/mL TGF-β2, 4 ng/mL TGF-β3, or 50 ng/mL BMP4 alone or in combination with 10 ng/mL TNF. Bottom panels show immunoblotting of MMP9 in CM and GAPDH in WCL. **E**. Immunoblotting with antibodies to phospho-SMAD1/5/8, BMP-signaling markers, and GAPDH using lysates from MDA-MB-231 cells treated with 50 ng/mL BMP4 for the indicated times. **F**. Gelatin zymography of 48-hour conditioned media from MDA-MB-231 cells, empty-vector control EGFP or dominant-negative TAK1-K63W (dnTAK1), that were treated with 2 ng/mL TGF-β1 and/or 10 ng/mL IL-1β. Fetal bovine serum (FBS) was used as a control for MMP9 and MMP2 activity.

To address the mechanism of the co-regulation of MMP9 by TGF-β and pro-inflammatory cytokines, we first examined the kinetics of this response. Assessment of MMP9 by zymography assays showed that co-treatment with TNF and TGF-β cytokines markedly induced secretion of pro-MMP9 at 24 hours in breast and lung cancer cells (Figure 3A-3B). Immunoblotting confirmed up-regulation of pro-MMP9 in 24-hr conditioned media (CM) in response to the cytokine co-treatment (Figure 3A-3B). MMP9 levels in whole-cell lysates were also up-regulated at 24 hours, although in A549 cells an increase in MMP9 was observed as early as 6 hours. The delay in secreted MMP9 is consistent with the known time-frame of MMP9 biosynthesis [39]. We also noted that the tested cytokines induced a 92kDa zymogen form of MMP9 while the tumor-fibroblast co-cultures produced an active 85kDa form of MMP9, suggesting an additional level of MMP9 regulation in the cell co-cultures.

Next, we asked whether co-regulation of MMP9 depends on TGF-β receptor activity and whether other TGF-β family cytokines can co-operate with TNF in this response. Co-treatment with TNF and TGF-β1 cytokines up-regulated levels of secreted MMP9, whereas this response was markedly reduced in the presence of the ALK5/TGFBR1 inhibitor SB525334 (Figure 3C). The ALK5/TGFBR1 inhibitor blocked SMAD signaling but did not affect TNF signaling (data not shown). Similar results were obtained in assays with A549 cells (Supplementary Figure 4B).

We then addressed the specificity of MMP9 co-regulation by TNF and TGF-β-family cytokines. Three TGF-β-type proteins activate ALK5/TGFBR1 signaling while bone-morphogenic proteins (BMPs) act through a different set of receptors. Tumor cells were treated with TNF in combination with TGF-β-type proteins and BMP4. Immunoblotting and zymography assays showed that all three TGF-β-type proteins are capable of cooperation with TNF in the regulation of MMP9 (Figure 3D). In contrast, BMP4 did not regulate MMP9 (Figure 3D), although BMP4 readily induced phosphorylation of SMAD1/5, a BMP-specific signaling response (Figure 3E). We next tested whether IL-1β and TGF-β1 can cooperate in MMP9 regulation, and whether this response depends on TAK1, which is important for MMP9 regulation by TGF-β1 (17). Zymography assays revealed that treatment with IL-1β and TGF-β1 co-operatively enhanced MMP9 secretion in MDA-MB-231 empty-vector control cells, whereas this response was completely blocked in cells expressing a kinase-inactive TAK1-K63W mutant (dnTAK1, Figure 3F). Together these results revealed that TGF-β-type cytokines cooperate with TNF and IL-1β cytokines in the stimulation of MMP9 expression in tumor cells. This response depends on the TGF-β receptor and TAK1 activities.

### TAK1-IKK-RELA signaling contributes to expression of MMP9

**Figure 4:**
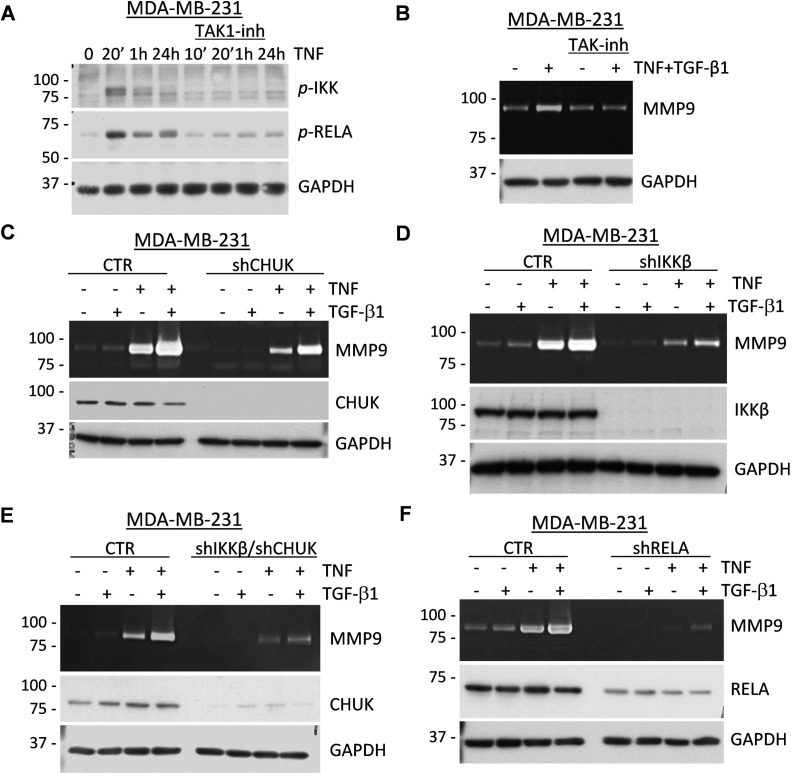
TAK1 signaling is essential for the induction of MMP9 by TNF and TGF-β cytokines **A**. MDA-MB-231 cells were treated for the indicated times with 10 ng/mL TNF ± 5μM (5Z)-7-Oxozeaenol, a TAK1 inhibitor. Whole-cell extracts were probed with antibodies to phosphorylated forms of IKK kinases or RELA/p65, and GAPDH, a loading control. **B**. Top panel shows gelatin zymography with 48-hour conditioned media from MDA-MB-231 cells co-treated with 2 ng/mL TGF-β1 and/or 10 ng/mL TNF ± 5μM (5Z)-7-Oxozeaenol. Bottom panel shows probing of whole-cell extracts for GAPDH. **C**.-**F**. Top panels show gelatin zymography of 48-hour conditioned media from MDA-MB-231 cells expressing shRNA to CHUK/IKKα (C), IKKβ (D), or both (E); or to RELA/p65 (F) treated with 2 ng/mL TGF-β1 and/or 10 ng/mL TNF. Whole-cell lysates were immunoblotted for the corresponding shRNA targets and GAPDH was used as a loading control.

To assess the mechanism of the co-operative regulation of MMP9 by TGF-β1 and TNF cytokines, we explored the TAK1 signaling pathway, which has been implicated in the activation of canonical NF-κB and MAPK signaling in response to TNF and IL-1β cytokines [40]. First, we validated activation of the NF-κB signaling axis. Ligation of TNF leads to the TAK1-mediated activating phosphorylation of IKK-β (Inhibitor of nuclear factor Kappa-B Kinase subunit beta) and IKK-α/CHUK which, in turn, phosphorylate RELA, a subunit of the canonical NF-κB transcription factor [40]. TNF stimulated phosphorylation of the IKK kinases and RELA/p65 (Ser536) and these events were blocked by the selective TAK1 inhibitor (5Z)-7-oxozeaenol [41] (Figure 4A). Zymography assays showed that the TAK1 inhibitor effectively blocked MMP9 induction by the TGF-β1 and TNF cytokines (Figure 4B). To assess the role of IKK kinases in MMP9 regulation, MDA-MB-231 cells were depleted of these kinases using small-hairpin (sh) RNA (Supplementary Figure 5A). Knockdown of CHUK moderately reduced expression of MMP9, while knockdown of IKK-β had a more prominent effect (Figure 4C-4D). Depletion of both kinases resulted in a nearly complete blockade of MMP9 regulation (Figure 4E and Supplementary Figure 5A). Finally, knockdown of RELA/p65 in MDA-MB-231 revealed an essential role of RELA in the regulation of MMP9 by TGF-β1 and TNF cytokines (Figure 4F and Supplementary Figure 5B). Together these results strongly support a critical role of the TAK1-IKK-RELA signaling axis in the cooperative regulation of MMP9 by TGF-β and TNF cytokines.

### TGF-β and TNF signaling crosstalk in tumor cells

**Figure 5:**
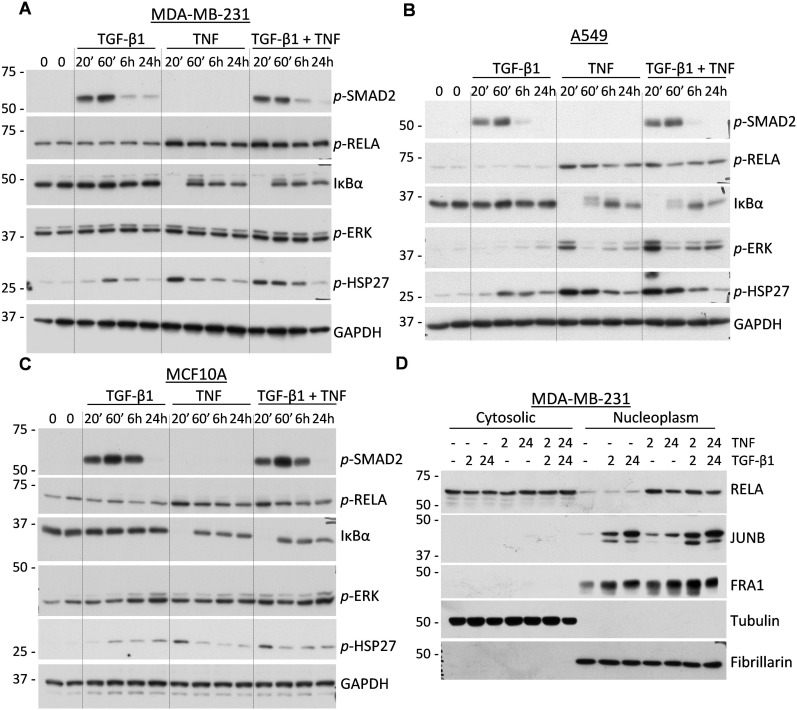
Regulation of signal transduction cascades in response to TGF-β1 and TNF **A**.-**C**. Immunoblot analysis of whole-cell lysates from MDA-MB-231, A549, and MCF10A cells treated with 2 ng/mL TGF-β1, 10 ng/mL TNF, or their combination. Membranes were probed with antibodies to phospho-SMAD2, phospho-RELA, phospho-ERK1/2, phospho-HSP27, IκBα, and GAPDH, a loading control. **D**. Immunoblotting of cytoplasmic and nuclear fractions of MDA-MB-231 cells treated with 2 ng/mL TGF-β1, 10 ng/mL TNF, or their combination for 2 and 24 hours. Membranes were probed with antibodies to RELA/p65, JUNB, and FRA1/FOSL1. Tubulin and fibrillarin were probed to validate efficient separation of the two fractions.

Given the essential role of the TAK1-IKK-RELA signaling axis in MMP9 regulation, we next asked whether TGF-β and TNF cytokines cooperate in signal transduction events leading to the activation of this axis. MDA-MB-231 and A549 cancer cells were treated with TGF-β1, TNF, or their combination and whole-cell lysates were probed for signaling markers. Treatment with TGF-β1 alone stimulated phosphorylation of receptor-associated SMAD2 in both cell lines, but did not induce phosphorylation of RELA (Figure 5A-5B). Likewise, TNF alone stimulated phosphorylation of RELA at Ser536 and degradation of IκBα, biomarkers of TNF signaling, while TGF-β1 had no effect on these TNF-induced signaling events. In cells co-treated with both cytokines, the dynamics and levels of these canonical signaling responses were essentially similar to those of cells treated with the individual cytokines (Figure 5A-5B). Accordingly, only TNF stimulated expression of NFKB2 (data not shown), a well-known downstream target of RELA [28]. Similar results were obtained in the non-tumor breast cell line MCF10A (Figure 5C).

MAPK signaling was evaluated by examining the phosphorylation levels of ERK1/2 and HSP27 (a target of p38MAPK). Levels of both markers were increased at 20 min in response to TNF and at 1 hour by TGF-β1 in all three cell lines (Figure 5A-5C). Co-treatments with the cytokines resulted in additive responses, suggesting that separate pools of the MAPK signaling molecules are involved in distinct signaling events.

To assess whether the cytokine signaling cascades converge at the level of nuclear translocation of NF-κB and/or AP1 transcription factors, we prepared nuclear and cytosolic fractions of MDA-MB-231 cells treated with TGF-β1, TNF, or their combination. Immunoblotting for tubulin and fibrillarin showed an effective separation of the cytoplasmic and nuclear fractions, respectively (Figure 5D). Probing for RELA revealed that TNF stimulated localization of RELA into the nucleoplasm, while TGF-β1 alone or in combination had no effect on this response (Figure 5D). NFKB2 levels were induced by TNF, but no nuclear translocation was observed for either the p100 or p52 forms (data not shown). Assessment of AP1 subunits showed that both the TGF-β and TNF cytokines increased the protein levels of JUNB and FRA1/FOSL1 in the nucleoplasm (Figure 5D). This is consistent with the regulation of JUNB by TGF-β cytokines [42]. Together, these findings argue that TGF-β and TNF cytokines co-stimulate the MAPK-AP1 signaling axis, promoting the nuclear accumulation of JUNB and FRA1, but do not co-operate in the canonical SMAD and NF-κB signaling events.

### The role of MAPK-AP1 signaling in the regulation of MMP9 by TGF-β and TNF cytokines

**Figure 6:**
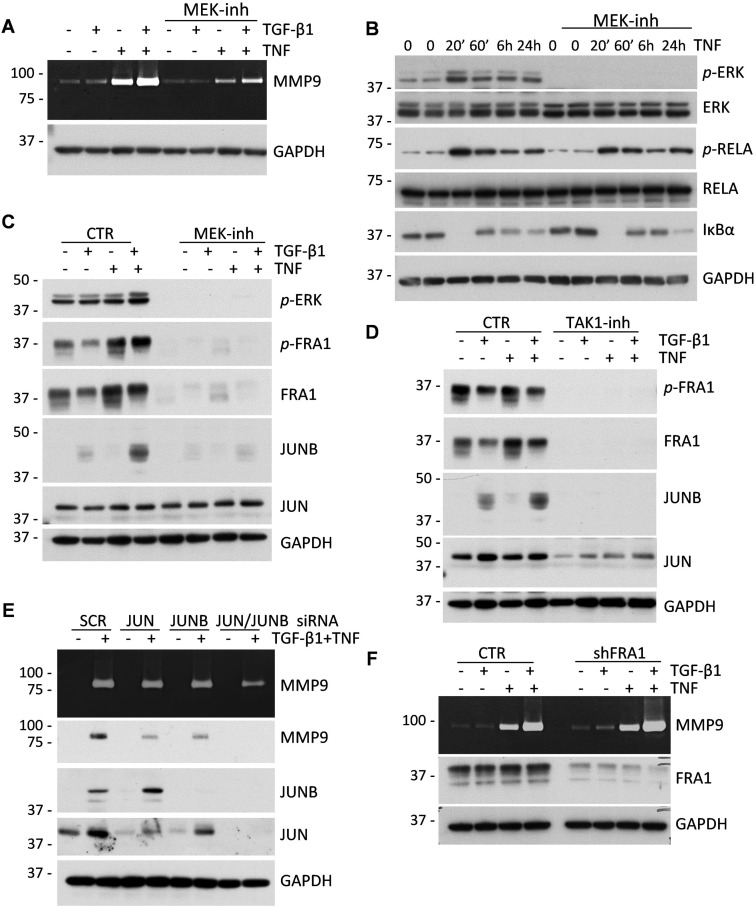
MAPK-AP1 signaling contributes to the up-regulation of MMP9 but FRA1 is dispensable for MMP9 expression **A**. Gelatin zymography of 48-hour conditioned media from MDA-MB-231 cells treated with 2 ng/mL TGF-β1 and/or 10 ng/mL TNF ± 5μM U0126, a MEK inhibitor. Lower panel shows probing for GAPDH in whole-cell lysates. **B**. Immunoblot analysis of TNF signaling in MDA-MB-231 cells treated with 10 ng/mL TNF ± 5μM U0126 for the indicated times. **C**. Immunoblotting of lysates from MDA-MB-231 cells treated with 2 ng/mL TGF-β1, 10 ng/mL TNF, or their combination ± 5μM U0126. GAPDH serves as a loading control. **D**. Immunoblot analysis of MDA-MB-231 cells treated with 2 ng/mL TGF-β1, 10 ng/mL TNF, or their combination ± 5μM TAK1 inhibitor, (5Z)-7-Oxozeaenol. **E**. Gelatin zymography of 48-hour conditioned media from MDA-MB-231 cells transfected with siRNA to JUN, JUNB or both and treated with 2 ng/mL TGF-β1, 10 ng/mL TNF, or their combination. Lysates were probed for MMP9, JUN, JUNB and GAPDH. **F**. Gelatin zymography of 48-hour conditioned media of MDA-MB-231 cells infected with lentiviruses for empty-vector control or shRNA to FRA1. Lower panels show immunoblotting for FRA1 and GAPDH.

We next asked whether MAPK-AP1 signaling contributes to the regulation of MMP9 by TGF-β and TNF cytokines. Treatment of MDA-MB-231 cells with cytokines in the presence of U0126, a selective MEK inhibitor, reduced the expression of MMP9 (Figure 6A) and the phosphorylation of ERK (Figure 6B), without an apparent effect on TNF-RELA signaling (Figure 6B). Furthermore, the MEK inhibitor completely blocked the up-regulation of total and phosphorylated FRA1 and total JUNB proteins by TGF-β and TNF cytokines (Figure 6C). Expression of JUN was not affected by the MEK inhibitor (Figure 6C). In comparison, the TAK1 inhibitor blocked the regulation of all three AP1 proteins, i.e. FRA1, JUN, and JUNB (Figure 6D), suggesting that TAK1 may regulate JUN *via* a MEK-independent pathway.

To define the role of these AP1 components in the cytokine-mediated regulation of MMP9, tumor cells were depleted of these proteins using RNA interference (Figure 6E-6F). Immunoblotting and zymography assays showed a moderate decline of MMP9 induction by depletion of JUN and JUNB alone, while depletion of both JUN and JUNB significantly reduced MMP9 expression in response to the cytokines (Figure 6E). In contrast to JUN/JUNB, depletion of FRA1 did not block regulation of MMP9 by the cytokines (Figure 6F). This result was further validated using siRNA in A549 cells (Supplementary Figure 6). Thus, the MAPK-AP1 signaling axis is likely to be responsible for the cooperative regulation of MMP9 by TGF-β and TNF cytokines; and JUN/JUNB are important AP1 components in this response.

### A critical role of tumor-derived MMP9 in tumor xenograft growth

**Figure 7:**
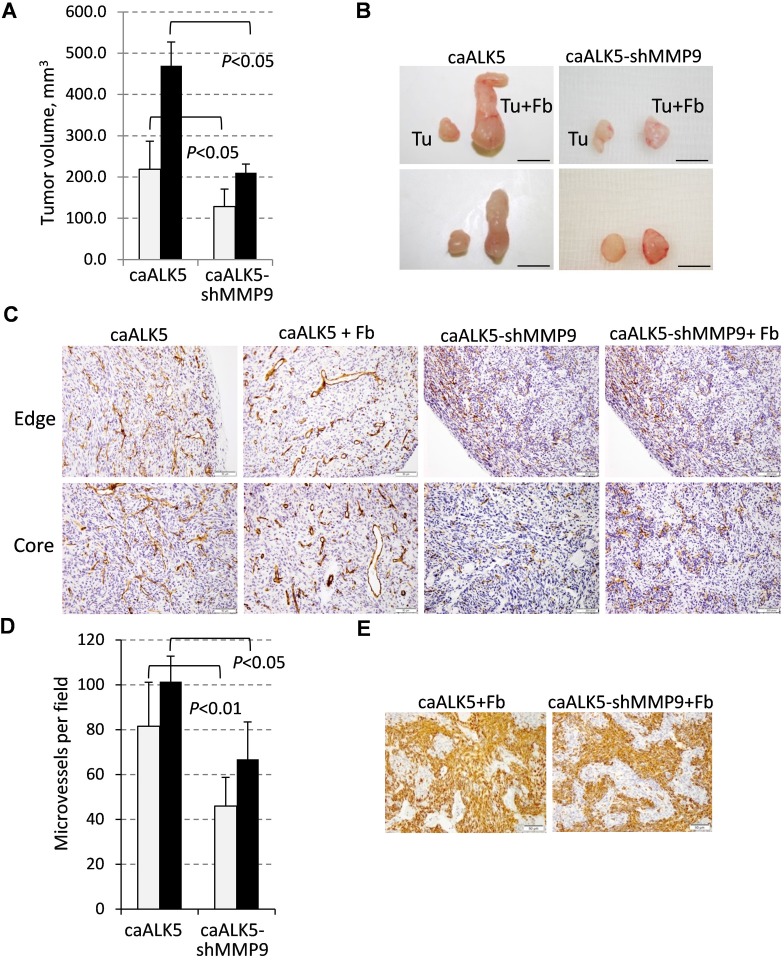
Fibroblast-mediated enhancement of tumor growth and vascularization depends on MMP9 **A**. Tumor volume at the endpoint of the xenograft study with MDA-MB-231 cells expressing constitutively active ALK5/TGFBR1 (caALK5) or caALK5 and shRNA to MMP9 ± 208F fibroblasts. **B**. Images of tumors excised at the endpoint of the study. **C**. CD31 staining in xenografts from study in (A). **D**. Quantification of microvessel density measured in six fields for each tumor section and presented as a mean number per field (0.2 mm^2^). **E**. Staining of rat fibroblasts with an antibody to prolyl 4-hydroxylase (rPH) in xenograft tumor sections. Images were taken at 200× magnification.

To address the role of tumor-derived MMP9 in tumor vascularization, we utilized MDA-MB-231 cells expressing constitutively-active ALK5/TGFBR1-T204D (caALK5) and MDA-MB-231-caALK5 cells depleted of MMP9 by shRNA [13]. Addition of fibroblasts to the MDA-MB-231-caALK5 xenografts significantly increased the tumor sizes at the end point of the study (Figure 7A-7B), similar to our findings with MDA-MB-231 cells (Figure 1A-1C). Depletion of MMP9 significantly reduced tumor growth in both the tumor alone and tumor-fibroblast groups (Figure 7A-7B). CD31 staining showed that tumor blood vessels were enlarged in the presence of fibroblasts at the tumor periphery as well as in the tumor core (Figure 7C). Depletion of MMP9 resulted in a marked reduction in blood vessel density, as well as eliminated large blood vessels in both xenograft groups (Figure 7C-7D). To rule out an effect of MMP9-depletion on fibroblasts, we examined the presence of rat fibroblasts in tumors using the rat specific marker rPH. Immunostaining detected rPH-positive cells only in tumor-fibroblast xenografts and depletion of MMP9 did not affect the presence of rat fibroblasts in the xenografts (Figure 7E). Assessment of TUNEL staining revealed the presence of dead cells in the core of tumor-alone xenografts while this staining was reduced in the tumor-fibroblast xenografts (data not shown), similar to the experiments with MDA-MB-231 cells (Supplementary Figure 1A). Together these findings show that fibroblasts enhance tumor vascularization and tumor-derived MMP9 plays a critical role in this response.

## DISCUSSION

The current study found that fibroblasts enhance breast carcinoma growth by promoting the tumor vasculature *via* the MMP9-dependent mechanism. Experiments with tumor-fibroblast co-cultures revealed that fibroblasts increase expression of TGF-β, TNF, and IL-1β cytokines in tumor cells, as well as stimulate TGF-β signaling. These findings are consistent with the metadata showing elevated levels of these cytokines and MMP9 and the presence of stroma in human breast carcinomas. Accordingly, secretion of MMP9 was significantly increased in tumor-fibroblast co-cultures and this effect was dependent on TGF-β signaling. Furthermore, co-treatment of tumor cells with TGF-β and TNF/IL-1β cytokines cooperatively stimulated expression of MMP9. Our study showed that the mechanism of this synergistic response did not involve a cross-activation of the canonical signaling pathways. Instead, the cytokines co-stimulated expression of the AP1 transcription factor components JUN and JUNB. Depletion of JUN and JUNB or disruption of TAK1-RELA signaling blocked the cytokine-mediated induction of MMP9 in tumor cells. Thus, our study revealed a previously unappreciated role of tumor-fibroblast interactions in stimulation of tumor vascularization, and showed an essential role of cytokine crosstalk in the cooperative up-regulation of the angiogenic driver MMP9.

Tumor-associated fibroblasts are thought to contribute to tumorigenesis by promoting recruitment of immune cells into the tumor by upregulating levels of chemokines and cytokines [3, 5]. Fibroblasts in breast carcinoma models increase levels of pro-inflammatory chemokines, including CCL2 and CCL7, which enhance infiltration of myeloid immune cells (macrophages, neutrophils) promoting tumor angiogenesis [5, 43]. Our study indicates that, fibroblasts act upon carcinoma cells to increase the expression of pro-angiogenic factors such as MMP9. Fibroblasts did not express appreciable MMP9 levels but rather enhanced production of MMP9 by carcinoma cells *via* cytokine signaling crosstalk. Thus, all three main cellular components of the breast TME contribute to the formation of tumor blood vessels, although the underlying mechanisms are different.

We explored the mechanism of MMP9 regulation in direct co-cultures of carcinoma cells and fibroblasts. These experiments revealed elevated levels of TGF-β cytokines and TGF-β signaling in co-cultures compared to cell monocultures (Figure 2A-2B). Our results are consistent with the increased expression of TGF-β and pro-inflammatory cytokines in both the tumor and stromal compartments of human breast carcinomas (Supplementary Figure 3). Testing whether TGF-β and pro-inflammatory TNF/IL-1β cytokines cooperate in the regulation of MMP9, we found that MMP9 expression was induced only in tumor cells, while fibroblasts expressed high levels of MMP2 (Figure 2E-2F). Testing the interaction of cytokines, we found that only TGF-β-type cytokines but not BMP-like cytokines cooperate with pro-inflammatory cytokines in the regulation of MMP9 (Figure 3D). Questions on how fibroblasts enhance cytokine expression and why TGF-β-type, but not BMP-like, cytokines cooperate with TNF in the regulation of MMP9 need further investigation.

Exploring the mechanism of MMP9 regulation by cytokine crosstalk in carcinoma cell lines, we found that this response requires TAK1 and both IKK kinases, as well as RELA/p65, a subunit of the canonical NF-κB transcription factor (Figure 4). Inhibition or knockdown of these signaling components reduced or completely blocked induction of MMP9 by the cytokines. Assessment of signal transduction revealed that TGF-β did not stimulate the TAK1-IKK-RELA signaling axis. Instead, TGF-β and TNF cytokines cooperated in regulation of the MAPK-AP1 signaling axis (Figures 5–6). Inhibition of MEK signaling reduced expression of MMP9 and blocked up-regulation of AP1-components FRA1 and JUNB, but did not affect expression of JUN (Figure 6C). TAK1 inhibition blocked expression of all three AP1 proteins (JUN, JUNB, and FRA1/FOSL1) (Figure 6D). JUN and JUNB may form homo- and hetero-dimeric AP1 transcription factor, while FRA1/FOSL1 can form heterodimers with either JUN or JUNB [44]. Knockdown of JUN or JUNB alone had only a moderate effect on MMP9, whereas depletion of both JUN-type proteins effectively reduced expression of MMP9, similar to the MEK inhibitor (Figure 6). In contrast, depletion of FRA1/FOSL1 had no effect on MMP9 regulation in two tested cell lines. Thus, JUN and JUNB contribute to the regulation of MMP9, while FRA1 is dispensable. The defined role of JUN/JUNB and RELA in the regulation of MMP9 by cytokine crosstalk is consistent with the presence of AP1 and NF-κB functional sites within the proximal promoter of *MMP9* [39]. In addition, both JUN proteins contribute to the TGF-β-SMAD transcriptional program of EMT [42], and can substitute each other in transcriptional responses [45]. Together, these findings argue that TGF-β and TNF/IL-1β cytokines cooperate in the regulation of MMP9 by stimulating the MAPK-AP1 (both cytokines) and TAK1-RELA (TNF) signaling axes.

Breast carcinomas frequently contain significant amounts of stroma which provides vascular support for tumor growth [46]. Our study reveals that fibroblasts can promote tumor vascularization by acting directly upon breast carcinoma cells to stimulate expression of pro-angiogenic MMP9. Consistent with our findings, it has been recently reported that tumor-associated fibroblasts accumulate in breast carcinomas with stroma deficient for *Cav1*, leading to enhanced tumor vascularization [47]. This response has been linked to mTOR signaling, although the contribution of the TAK1-MMP9 axis was not investigated. Fibroblasts can also enhance tumor infiltration by immune cells producing MMP9. Tumor angiogenesis is stimulated by the influx of neutrophils that release highly angiogenic pro-MMP9 free of TIMP1, the natural inhibitor of MMP9, or M2 macrophages expressing high levels of pro-MMP9 and low levels of TIMP1 [48, 49]. In addition, inflammatory mast cells (50) and immune-suppressive myeloid-derived cells may also contribute to angiogenesis *via* an MMP9-dependent mechanism [9]. Taking into account that fibroblasts are the predominant cell type in breast cancer stroma [3, 4], tumor-fibroblast crosstalk represents a critical target for therapeutic intervention of tumor angiogenesis, growth, and metastasis.

**Figure 8:**
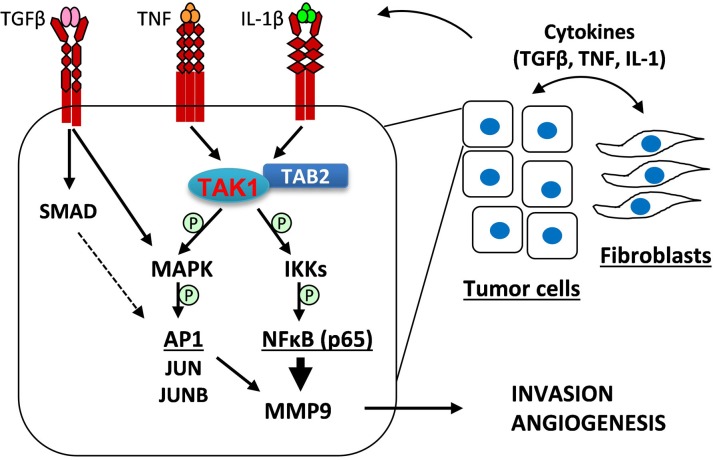
A model of tumor-fibroblast interactions in the regulation of MMP9 expression Tumor-fibroblast interactions increase levels of TGF-β, TNF, and IL-1β cytokines, which act upon tumor cells to cooperatively activate MAPK signaling as well as cytokine-specific canonical signaling: TGF-β stimulates SMAD2/3 signaling while TNF and IL-1β cytokines induce TAK1-RELA signaling. MAPK signaling regulates expression of FRA1 and JUNB, AP1 transcription factor subunits. AP1 and RELA transcription factors cooperatively regulate expression of MMP9, which promotes tumor invasion and angiogenesis.

In summary, the current study provides evidence that tumor-associated fibroblasts promote tumor angiogenesis by stimulating expression of the angiogenic driver MMP9 in breast carcinoma cells (Figure 8). Fibroblasts increase expression of TGF-β, TNF, and IL-1β cytokines which synergistically stimulate production of MMP9. Mechanistically, TGF-β and TNF/IL-1β cytokines cooperate in activation of the MAPK-AP1 (JUN/JUNB) signaling axis which, acting together with the TAK1-RELA axis, up-regulates expression of MMP9. Among the remaining questions is the mechanism underlying up-regulation of the cytokines which stimulate MMP9. It would also be important to determine whether tumor-fibroblast interactions regulate other pro-angiogenic factors in the MMP9-driven angiogenesis. Our mechanistic studies suggest therapeutic approaches blocking the pro-tumorigenic activity of fibroblasts, such as targeting TAK1 and IKK kinases or using neutralizing antibodies to TGF-β.

## MATERIALS AND METHODS

### Cytokines, antibodies and other reagents

Recombinant human TGF-β1 (Cat# 240-B/CF), TGF-β2 (Cat# 302-B2/CF), TGF-β3 (Cat# 243-B3/CF) and BMP4 (Cat# 314-BP/CF) were obtained from R&D Systems (Minneapolis, MN); recombinant human TNFα (Cat# CYT-223) was from ProSpec-Tany TechnoGene Ltd (Rehovot, Isreal); and recombinant human IL-1β (Cat# 200-01B) was from PeproTech (Rocky Hill, NJ). Antibodies for: GAPDH, IKKα/β, IκBα, JunB, Fibrillarin, FRA1/FOLS1 and c-Jun were from Santa Cruz Biotechnology, Inc. (Santa Cruz, CA); phospho-IKKα/β, phospho-RELA/p65, phospho-Smad2, phospho-p44/42 MAPK (ERK1/2), phospho-HSP27, phospho-FRA1/FOLS1, IKKα/CHUK, RELA/p65, MMP9, phospho-Smad1/Smad5/Smad8 and Stat3 were from Cell Signaling Technology (Danvers, MA); α-Tubulin was obtained from Sigma-Aldrich (St. Louis, MO); and cIAP2 was from Enzo Life Sciences (Farmingdale, NY). For more details on antibodies please see Supplementary Table 1. Goat anti-Rabbit IgG (H+L)-Horseradish Peroxidase (HRP) (Cat# 170-6515) and goat anti-Mouse IgG (H+L)-HRP (CAT# 170-6516) secondary antibodies were from BIO-RAD Laboratories (Hercules, CA); goat anti-Rat IgG (H+L) HRP conjugate (Cat# 31470) was from Pierce Biotechnology (Rockford, IL). Inhibitors of TAK1, (5Z)-7-Oxozeaenol (Cat# 499610), and MEK, U0126 (Cat# 662005), were from EMD Millipore (Billerica, MA); and the inhibitor of TGFβRI, SB525334 (Cat# 16281) was from Cayman Chemical Company (Ann Arbor, MI). Plasmids encoding short hairpin RNA (shRNA) to human CHUK/IKKα, IKBKB/IKKβ, RELA/p65, FRA1/FOSL1 and MMP9 in the pGIPZ lentiviral vector were from the shRNA Core Resource at Roswell Park Cancer Institute (RPCI), supported by the NCI Cancer Center Support Grant CA016056 to the RPCI. Short interfering RNA (siRNA) to human c-Jun (Cat# sc-29223) and human FRA1 (Cat# sc-35405) were from Santa Cruz Biotechnology, Inc. and siRNA to human JUNB (Cat# SASI_Hs01_00204771) was from Sigma-Aldrich.

### Cell culture

Human metastatic breast carcinoma cell line MDA-MB-231, rat embryonic fibroblast cell line 208F, human embryonic lung fibroblast cell line WI38, human lung carcinoma cell line A549, human luminal breast carcinoma cell line MCF7 and human mammary epithelial cell line MCF10A were obtained from American Tissue Culture Collection (ATCC) (Manassas, VA) and cultured as recommended by ATCC. MDA-MB-231-caALK5 and MDA-MB-231-caALK5-shMMP9 cells expressing constitutively active TGFBR1/ALK5-T204D mutant and shRNA to MMP9 are described elsewhere [13]. MDA-MB-231-dnTAK1 cells expressing kinase inactive TAK1-K63W are previously described [17, 18].

### Retroviral infection

Cells were infected in the presence of 6 μg/ml polybrene (Cat# sc-134220; Santa Cruz) with amphotropic retroviruses produced in Phoenix cells. Viral supernatants were collected each day for 3 days, filtered and stored in aliquots at -80°C. EGFP-positive cells were selected by FACS.

### Lentiviral infection

Lentiviruses produced by HEK293T cells were used to infect cells by centrifugation in the presence of 4 μg/mL polybrene (Santa Cruz). EGFP-positive cells were selected in the presence of 2 μg/mL puromycin (Cat# P7255; Sigma-Aldrich).

### Short interference RNA

Cells were transfected with RNA duplexes using Lipofectamine 2000 (Cat# 11668027; Invitrogen, Thermo Fisher Scientific; Waltham, MA) following the manufacturer's protocol. Cells were seeded and grown in the absence or presence of 2 ng/mL TGF-β1 and/or 10 ng/mL TNFα for 48 hours followed by gelatin zymography and immunoblotting.

### Animal studies using a subcutaneous xenograft model

Female SCID/CB17 mice, 6 weeks of age, were obtained from a colony of SCID/CB17 mice bred and maintained at the Department of Laboratory Animal Resources (DLAR) facility at RPCI. Animals were kept 5 mice per cage in microinsulator units and provided with food and water *ad libitum* according to a protocol approved by the Institute Animal Care and Use Committee (IACUC) at RPCI. The facility has been certified by the American Association for Accreditation of Laboratory Animal Care and in accordance with current regulation and standards of the US Department of Agriculture and the US Department of Health and Human Services. Exponentially growing breast cancer cells (1.5 × 10^6^) in 0.1 mL 50% sterile phosphate buffered solution (PBS): 50% reduced growth factor basement membrane extract type 2 (BME) were injected with a 27G needle into the left flank of 8 week old female SCID mice. Breast cancer cells (1.5 × 10^6^) mixed in a 3:1 ratio with exponentially growing fibroblast cells (0.5 × 10^6^) in 0.1 mL 50% PBS: 50% BME were injected into the right flank of the same 8 week old female SCID mice. Primary tumor growth was monitored by measuring tumor diameters with electronic calipers every 2-3 days after the appearance of palpable tumors. Volumes were calculated using the formula (length) × (width)^2^/2. After 9 weeks, mice were sacrificed and tumors were collected for histological analysis at the RPCI Pathology Core Facility.

### Immunohistochemistry

Tumor sections were fixed immediately after excision in 10% (v/v) formalin or, for CD31 staining, Zinc Fixative (Cat# 550523; BD Biosciences, NJ) before embedding in paraffin. Before immunostaining, conventional H&E-stained sections were prepared for general histopathological evaluation. For CD31 staining, rat anti-mouse primary antibody to CD31 (Cat#550274, BD Biosciences) and biotinylated secondary anti-rat antibody (BD Biosciences) were used as described in [13]. Analysis of microvessel density was performed as described in [51]. Briefly, tumor sections were scanned at 100× magnification for areas containing the highest number of discrete CD31-positive microvessels (microvessel hot spots). Necrotic and immediately adjacent areas where microvessels are sparse were excluded from counting. Rat fibroblasts were detected by staining tumor sections with an antibody to the rat fibroblast specific marker prolyl 4-hydroxylase (6-9H6) (NBP2-33342; Novus Biologicals; Littleton, CO). CD31-positive (brown stain) vessels were counted at 400× magnification in 8 fields of each tumor section. Results were presented as mean number of microvessels/field (0.2 mm^2^) ± standard deviation. The luminal size of blood vessels was evaluated on slides immunostained for CD31 using a DP26 Olympus microscope camera attached to a BX46 Olympus microscope and cellSens Standard 1.11 imaging software by Olympus. At low magnification, the areas with the largest blood vessels in each case were identified and the diameters of five largest vessels in each of five microscopic fields were measured at 400× magnification in five tumors from each group. The results were presented as the mean number of diameters in each group ± standard deviation.

### Apoptosis staining

TUNEL staining was used for *in situ* detection of apoptotic cells in paraffin sections of tumor tissue using the ApopTag Plus Peroxidase *In Situ* Apoptosis Detection Kit (Cat# S7101; Chemicon, EMD Millipore) following the manufacturer's recommendations. Cells were examined in 5 random fields using light microscopy at 400× magnification.

### *In vitro* co-culture

Tumor cells were seeded 4.5 × 10^5^ cells per well in a 6-well plate individually or in combination with fibroblasts in a 3:1 ratio. Fibroblasts were seeded 1.5 × 10^5^ cells per well individually or in combination with tumor cells. Cells were left overnight to attach. The following day, cells were washed 3 times with PBS and 700 μL serum-free media was added to each well. After incubation for 48, 72 or 96 hours (depending on experiment) conditioned media and whole-cell lysates were collected and analyzed by zymography and immunoblotting.

### Gelatin zymography assay

Conditioned media from cells untreated or treated with cytokines and/or inhibitors in serum-free IMEM or DMEM was collected, centrifuged and concentrated as needed. SDS-PAGE gels were co-polymerized with gelatin at a final concentration of 1 mg/mL. After electrophoresis (120V, ~1.5 hours), the gels were renatured in 2.5% Triton X-100 and incubated in development buffer (0.05M TrisHCl pH 7.4, 5mM CaCl_2_, 0.2M NaCl, 0.02% NaN_3_) at 37°C with agitation for 18 hours. Gels were stained with Coomassie solution (0.5% (w/v) Coomassie Blue R250, 5% (v/v) methanol, 10% (v/v) acetic acid) for 2 hours followed by incubation in destaining solution (20% (v/v) methanol, 10% (v/v) acetic acid). Gelatinase activity is seen as a transparent band on a blue background.

### Immunoblot analysis

A detailed description of immunoblotting has been reported elsewhere [17, 18]. Briefly, whole-cell lysates were collected using NP40 Lysis Buffer (0.88% NP-40, 132 mM NaCl, 44 mM Hepes, 8.8 mM NaF, 2 mM sodium orthovanadate) supplemented with 1 mM PMSF and 1X Protease Inhibitor Cocktail (Cat# 11836153001; Roche; Basel, Switzerland). Prior to lysis, cells were grown to 70-80% confluency and treated with 2 ng/mL TGF-β1 and/or 10 ng/mL TNFα for indicated times. Inhibitors were added 1 hour prior to cytokine treatment. Protein concentrations were measured using the Bio-Rad DC Protein Assay according to the manufacturer's instructions. Proteins were resolved using 10% or 12.5% SDS-PAGE and transferred to nitrocellulose membranes (Cat# 162-0112; BIO-RAD). Transfer was validated by Ponceau S staining. Membranes were blocked with 5% milk for 1 hour at room temperature (RT) then incubated with the primary antibody in 5% milk or 3% BSA overnight at 4°C. After washing, membranes were incubated with secondary antibodies in 5% milk for 1 hour. Protein bands were visualized using ECL chemiluminescent reagent (Cat# 32209; Pierce).

### Nuclear and cytosolic protein collection

Cells were cultured in 10 cm dishes and treated with cytokines for the indicated times. Cells were collected in 1 mL ice-cold PBS by centrifugation. Cells were resuspended in hypotonic buffer (8.8 mM Hepes, 8.8 mM KCl, 0.88 mM EDTA, 0.88 mM EGTA, 0.35% NP40, 44 mM NaF, 1 mM PMSF, 2 mM Sodium Orthovanadate, 1 mM DTT and 1X Protease Inhibitor Cocktail (Roche)) and incubated for 10 minutes on ice followed by centrifugation at 4°C. The cytosolic fractions (supernatant) were collected after two subsequent centrifugations. The original pellet was washed with hypotonic buffer followed by the dropwise addition of high salt buffer (8.8 mM Hepes, 8.8 mM KCl, 0.88 mM EDTA, 0.88 mM EGTA, 44 mM NaF, 1 mM PMSF, 2 mM Sodium Orthovanadate, 1 mM DTT and 1X Protease Inhibitor Cocktail). Tubes were rotated for 30 min at 4°C, and the nuclear fractions (supernatant) were separated from cell debris by centrifugation at 4°C. Protein concentrations were measured and immunoblotted as described above. Extracts were stored at -80°C.

### qPCR analysis

Cells were incubated in media containing 5% serum for at least 4 hrs prior to treatment with cytokines. Inhibitors were added 1 hr prior to cytokine treatment. RNA extraction was performed using TRIzol Reagent (Cat# 15596-026; Invitrogen). cDNA samples were prepared using M-MLV RT (Cat# M1701; Promega; Madison, WI), and then were amplified using 5X HOT FIREPol EvaGreen qPCR Mix Plus (ROX) (Cat# 08-24-00001; Solis Biodyne, Tartu, Estonia) in the Applied Biosystems StepOnePlus Real-Time PCR System. Primer sequences are as follows: TGFB1, RefSeq NM_000660 (Forward: GCTGCTGTGGCTACTGGTGC, Reverse: CATAGATTTCGTTGTGGGTTTC); TGFB2, RefSeq NM_001135599 and NM_003238 (Forward: CCCCACATCTCCTGCTAA, Reverse: GTGTATCCATTTCCACCCTA); 5S rRNA (Forward: GGCCATACCACCCTGAACGC, Reverse: AGCCTACAGCACCCGGTATT).

### Statistical analysis

Data in each experiment was compared using the Student's *t* test. Statistical significance was achieved when *P* < 0.05.

## SUPPLEMENTARY FIGURES AND TABLE





## Abbreviations

TGF-β: transforming growth factor beta
TAK1: TGF-beta activated kinase 1
MAPK: mitogen-activated protein kinase
FRA1: Fos-related antigen-1
MMP9: matrix metalloproteinase-9
TNF: Tumor necrosis factor
TAFs: Tumor-associated fibroblasts
