# Supplementary material for: Tumor-fibroblast interactions stimulate tumor vascularization by enhancing cytokine-driven production of MMP9 by tumor cells
Source: Oncotarget. 2017 Mar 8;8(22):35592–608. doi: 10.18632/oncotarget.16022 (PMC5482601; doi:10.18632/oncotarget.16022)
Supplement: Supplementary file 1 [file oncotarget-08-35592-s001.pdf]

Tumor-fibroblast interactions stimulate tumor vascularization by enhancing cytokine-driven production of MMP9 by tumor cells

Supplementary Material

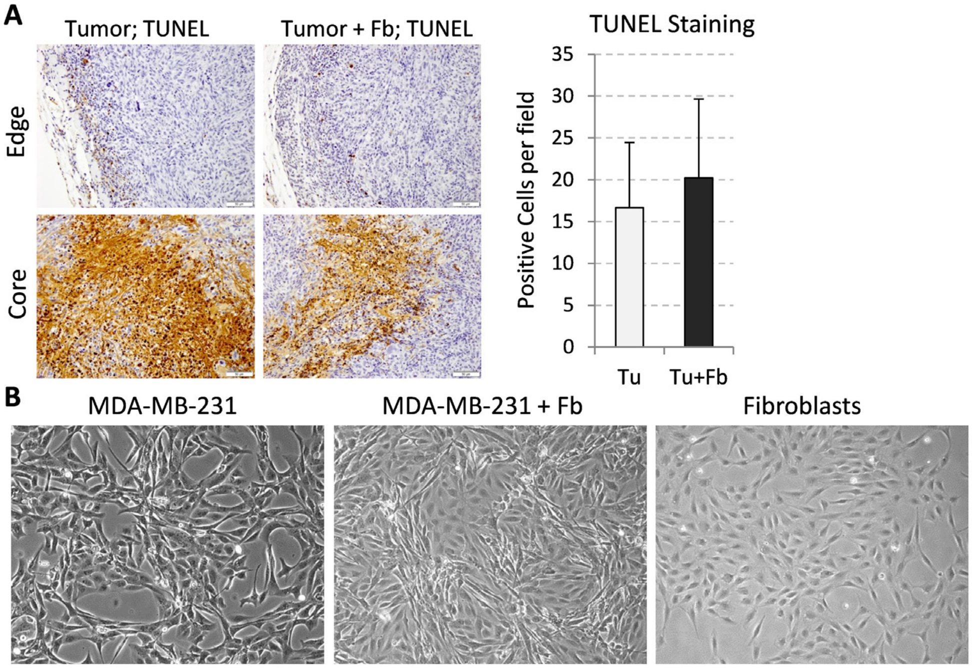

Supplementary Figure 1: (A) TUNEL staining of xenografts. TUNEL staining at the periphery (edge) and core of the tumor or tumor-fibroblast xenografts. Images were taken at 200× magnification. TUNEL-positive cells at the periphery from three fields in three tumors per group were counted and presented as a graph of the mean number of positive cells per field. (B) Images of in vitro co-cultures. Phase-contrast images of breast cancer MDA-MB-231 cells and rat 208F fibroblasts alone and in direct co-cultures. Images were taken at 200× magnification.

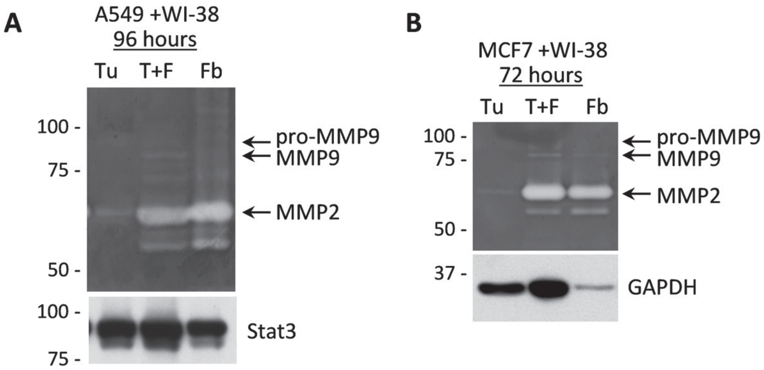

Supplementary Figure 2: (A) Gelatin zymography (top panel) with 96-hour conditioned media from A549 human lung cancer cells (Tu), WI-38 human fibroblasts (Fb), or their co-culture in a ratio of 3:1 (T+F). Total Stat3 in whole-cell extracts was used as a loading control. (B) Gelatin zymography with 72-hour conditioned media from MCF7 cells (Tu), human WI-38 fibroblasts (Fb), or their co-culture in a ratio of 3:1 (T+F). Total GAPDH in whole-cell extracts was used as a loading control.

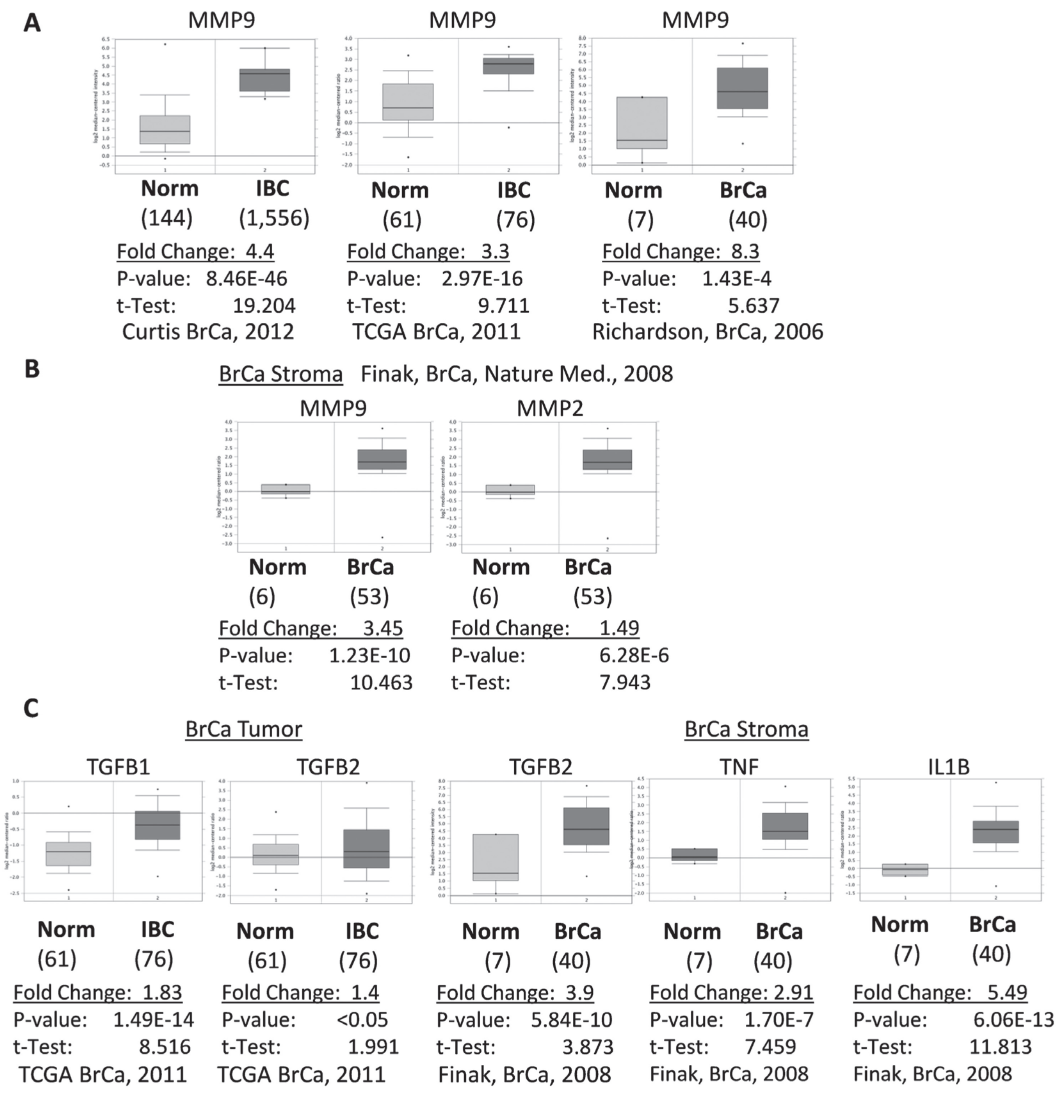

Supplementary Figure 3: Expression levels of MMP9 and cytokines in normal breast and breast carcinomas were obtained using the Oncomine database (www.oncomine.org).(A) MMP9 levels in breast carcinomas and normal breast: Curtis Breast (Nature, 2012), TCGA BrCa (2011), and Richardson Breast, (Cancer Cell, 2006). In the latter study total RNA was prepared from tumor sections using laser capture microdissection (LCM) followed by gene expression profiling using the Affymetrix platform. (B) MMP levels in the stroma adjacent to invasive breast carcinomas (50 ductal and 3 lobular carcinomas) obtained using the data from Finak Breast (Nature Medicine, 2008). (C) Expression of cytokines in breast cancer from TCGA BrCa (2011) and in the stroma adjacent to invasive breast carcinomas or normal tissues from Finak Breast (Nature Medicine, 2008).

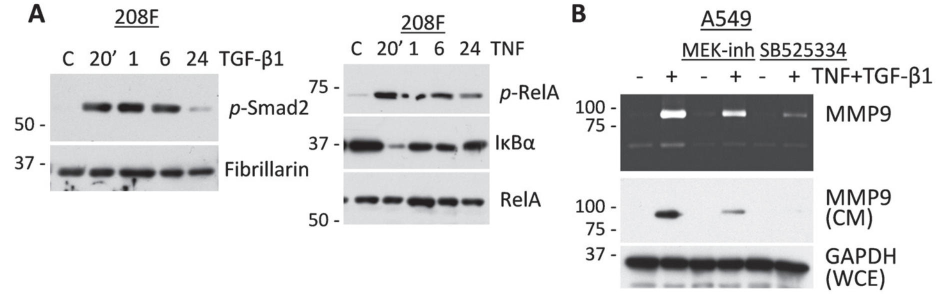

Supplementary Figure 4: (A) Cytokine signaling in fibroblasts. Whole-cell extracts from 208F fibroblasts treated with 2 ng/mL TGF-β1 or 10 ng/mL TNF for the indicated times were immunoblotted for phospho-Smad2, phospho-p65/RelA and IκBα. Fibrillarin or RelA were used as a loading control. (B) TGF-β signaling is important for MMP9 activity and secretion. Top panel shows gelatin zymography of 48-hour conditioned media from A549 cells treated with 2 ng/mL TGF-β1 and 10 ng/mL TNF ± 5μM U0126 or 10 μM SB525334, an ALK5 inhibitor. Bottom panels show immunoblotting of MMP9 in conditioned media (CM) or GAPDH in whole-cell extracts (WCE).

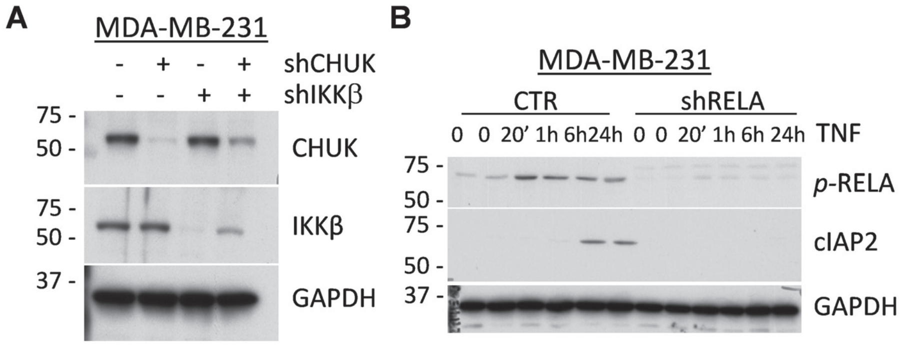

Supplementary Figure 5: Confirmation of shRNA knockdowns in MDA-MB-231 cells.(A) Immunoblotting of CHUK/IKKα, IKKβ and GAPDH (loading control) in whole-cell lysates from MDA-MB-231 cells infected with lentiviruses encoding shRNA to CHUK, IKKβ, or their combination. (B) Immunoblotting with antibodies to phospho-RELA/p65, phospho-ERK1/2, IκBα, and cIAP2/BIRC3 in whole-cell lysates of MDA-MB-231 cells infected with lentivirus encoding shRNA to RELA/p65 and treated with 10 ng/mL TNF. GAPDH is a loading control.

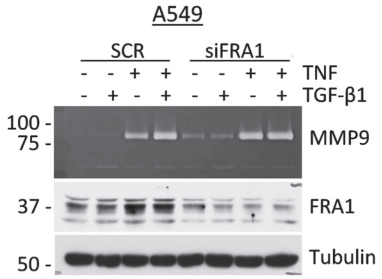

Supplementary Figure 6: Knockdown of FRA1/FOSL1 does not block induction of MMP9. A549 cells transfected with siRNA to scramble-control or FRA1 and treated with 2 ng/mL TGF-β1, 10 ng/mL TNF, or their combination for 48 hours. Top panel shows gelatin zymography with 48-hour conditioned media. Bottom panels show immunoblotting for FRA1 and α-tubulin, a loading control, in whole-cell extracts.

Supplementary Table 1: Detailed Antibody Information

Antibodies:

Antibody	Company	Catalog #
IKKα/CHUK	Cell Signaling	2682
MMP9 (D6O3H) XP	Cell Signaling	13667
NF-κB p65/RELA (D14E12) XP	Cell Signaling	8242
Stat3	Cell Signaling	9132
phospho-FRA1 (Ser265) (D22B1)	Cell Signaling	5841
phospho-HSP27 (Ser82)	Cell Signaling	2401
phospho-IKKα/β (Ser176/180) (16A6)	Cell Signaling	2697
phospho-NF-κB p65/RELA (Ser536) (93H1)	Cell Signaling	3033
phospho-p44/42 MAPK (ERK1/2) (Thr202/Tyr204) (20G11)	Cell Signaling	4376
phospho-Smad1 (Ser463/465)/Smad5 (Ser463/465)/Smad8 (Ser426/428)	Cell Signaling	9511
phospho-Smad2/3 (Ser465/467) (138D4)	Cell Signaling	3108
cIAP2/BIRC3 (16E-6-3)	Enzo Life Sciences	ALX-803-341
c-Jun (H-79)	Santa Cruz	sc-1694
Fibrillarin (H-140)	Santa Cruz	sc-25397
Fra1/FOLS1 (R-20)	Santa Cruz	sc-605
GAPDH (FL-335)	Santa Cruz	sc-25778
IκBα (C-21)	Santa Cruz	sc-371
IKKα/β (H-470)	Santa Cruz	sc-7607
JunB (N-17)	Santa Cruz	sc-46
α-Tubulin	Sigma-Aldrich	T6074
